# High-frequency ultrasound with superb microvascular imaging: a potential tool for ultrasound assessment in patients with giant cell arteritis

**DOI:** 10.3389/fmed.2024.1431385

**Published:** 2024-07-10

**Authors:** Johan Skoog, Christina Svensson, Per Eriksson, Christopher Sjöwall, Helene Zachrisson

**Affiliations:** ^1^Department of Clinical Physiology and Department of Health, Medicine and Caring Sciences, Linköping University, Linköping, Sweden; ^2^Department of Biomedical and Clinical Sciences, Division of Inflammation and Infection/Rheumatology, Linköping University, Linköping, Sweden

**Keywords:** giant cell arteritis, color duplex ultrasound, large vessel vasculitis, superb microvascular imaging, neovascularization

## Abstract

**Objective:**

The objective of this study was 2-fold: first, to evaluate whether superb microvascular imaging (SMI) could be used to visualize neovascularization in temporal arteries, and, second, to evaluate the diagnostic performance of high frequency ultrasound with SMI using an extended protocol in patients with suspected giant cell arteritis (GCA).

**Methods:**

This retrospective study comprised 120 patients consecutively examined with an extended CDU protocol (temporal, facial, axillary, subclavian, brachiocephalic, and carotid arteries) between 2020 and 2022. Of all patients, 107 had no previous GCA diagnosis and 13 had a previous GCA diagnosis. SMI was used to evaluate neovascularization in the temporal arteries. Arteritis were characterized as low- or medium-echogenic, homogeneous wall thickening, with or without a positive compression sign in the temporal arteries. The Halo count, i.e., the number of temporal and axillary artery segments with signs of arteritis, was evaluated. The reference was clinically diagnosed GCA confirmed after ≥6-month follow-up.

**Results:**

Of the eligible 107 patients with new suspected GCA, 33 (31%) received a clinical GCA diagnosis. Neovascularization was detected in 14 patients (43%). Patients with neovascularization displayed a higher halo count [median 6 (25th−75th percentile 4.75–7) vs. 3 (2-4-4), *p* = 0.005]. CDU of only the temporal arteries showed sensitivity and specificity (95% confidence intervals) of 94% (80–100%) and 100% (95–100%), respectively. The addition of extra-cranial arteries increased the sensitivity to 100%. Of the 13 patients investigated for suspected relapse, three had a clinically confirmed relapse. One of them displayed neovascularization together with other signs of inflammation.

**Conclusions:**

We show for the first time that inflammatory neovascularization of the temporal arteries can be detected by SMI. Neovascularization is associated with a more-widespread cranial disease. The value of neovascularization should be further investigated, especially for the detection of GCA relapse.

## Introduction

Giant cell arteritis (GCA) is a systemic vasculitis that mainly targets larger arteries (1). Temporal artery biopsy (TAB) has long been regarded as the gold standard for diagnosing GCA. However, the latest recommendations from European Alliance of Associations for Rheumatology (EULAR) are to use color duplex ultrasound (CDU), if available, as the initial diagnostic modality (2). Recently, several studies have validated CDU including the temporal and axillary arteries, for the diagnosis of GCA (2–4). Data for more extended CDU protocols that include additional extra-cranial vessels are scarcer but have shown improved sensitivity with retained high specificity for GCA (5–7). During the past few years, ultrasound equipment has been refined. Modern high-frequency probes that give higher resolution are increasingly available. Furthermore, novel ultrasound imaging modalities, such as superb microvascular imaging (SMI), have been developed (8). SMI is a technology that is based on an algorithm that identifies and separates tissue movements (clutter) from low-flow components. Conventional Doppler identifies high velocity arterial blood flow, whereas SMI identifies low-velocity blood flow, enabling assessments of the micro-circulation (8). This seems interesting from the pathophysiological point-of-view because ingrowth of the vasa vasorum and extension of neovascularization into the media have been associated with inflammation in patients with GCA (9–11). SMI has been used as an inflammatory marker in Takayasu's arteritis (TAK), where neovascularization in the vessel wall has been associated with active TAK disease (12–14). Vessel wall vascularisation with SMI in cases of active TAK has also been shown to correspond to fluorodeoxyglucose (FDG) uptake in positron emission tomography (PET) (8). However, only a few case reports have evaluated SMI in patients with suspected GCA, and the temporal artery has not been investigated (15, 16). The objective of this study was 2-fold: first, to evaluate whether superb microvascular imaging (SMI) could be used to visualize neovascularization in temporal arteries, and, second, to evaluate the diagnostic performance of high frequency ultrasound with SMI using an extended protocol in patients with suspected giant cell arteritis (GCA).

## Materials and methods

### Study population

This retrospective study comprised 120 patients who were consecutively examined with CDU at the Department of Clinical Physiology, Linköping, Sweden, between October 2020 and April 2022. Of these 120 patients, 13 had a previous GCA diagnosis in which CDU was conducted as a follow-up examination, and 107 patients had no previous GCA diagnosis and CDU was performed due to clinically suspected GCA. The diagnosis of GCA was based on both the CDU results and clinical parameters (17). Patients were classified as having GCA if the 1990 American College of Rheumatology (ACR) criteria were satisfied (18), and/or if the patients had the typical ultrasound picture of arteritis characterized by low- or medium-echogenic, homogeneous, wall thickening combined with increased levels of CRP and/or higher erythrocyte sedimentation rate (ESR) and regression of the initial symptoms after treatment with corticosteroids. Digital medical records at least 6 months after the CDU were reviewed by an experienced rheumatologist (P.E.), not responsible for the clinical care of the patients. Final clinical diagnosis of arteritis was assessed based on the re-evaluation of all digital medical records comprising both clinical and laboratory data. However, the patients were treated by different physicians and a standardized clinical protocol was not used. The 6-month clinical follow-up used as the reference diagnosis in the present study is commonly applied in studies of GCA (19, 20). Patients with clinically suspected GCA were excluded if they (i) died or migrated within 6 months after the CDU; or (ii) were treated with high doses of steroids more than 2 months preceding the CDU.

### CDU assessment

The Canon Aplio i800 (Canon Medical Systems, Tochigi, Japan) high-frequency ultrasound system with linear transducer i11LX3 and hockeystick transducer i22LH8 were used for the ultrasound measurements. The protocol has previously been described in detail, also including extra-cranial vessels (7, 21–23). Additionally, color SMI was employed to scan the temporal arteries. The SMI settings were configured with a velocity scale of 1.1 cm/s, a color frequency of 12 MHz, a color filter set to 4, and a frame rate of 56 fps. In brief, bilateral examinations of the three branches of the temporal artery (common superficial artery, parietal, and frontal branches) and the facial artery, as well as the axillary, subclavian, brachiocephalic and carotid arteries were conducted. The intima-media thickness (IMT) was measured in the common superficial temporal, axillary, subclavian and carotid arteries. Atherosclerotic plaques were evaluated and defined as focal areas in the vessel wall where IMT demonstrated an increase of either 0.5 mm or 50% compared to the IMT in the adjacent wall. One experienced vascular technologist performed the CDU examinations as part of a standardized routine examination. The same vascular technologist and one physician reviewed the CDU examinations.

### Interpretation of inflammation in the CDU assessments

Increased IMT with low- or medium-echogenic, circumferential, homogeneous wall thickening in at least one vessel with or without a positive compression test were considered as typical signs of arteritis. In this study, SMI was used to visualize neovascularization, which was considered to be indicative of active inflammation (8). Furthermore, low- or medium-echogenic areas outside the vessel wall, as well as sub-intimally, were interpreted as inflammatory oedema. Increased IMT with hyper-echogenic areas was assumed to represent long-standing inactive arteritis (24). However, a mixture of hyper-echogenic and low-/medium-echogenic wall thickening may be observed in cases of relapsing arteritis. The number of affected arteries was assessed, and the halo count (between 0 and 8) was calculated for the temporal an axillary arteries in accordance with the report of van der Geest et al. (20).

### Statistical evaluation

Data are presented as numbers and percentages or median with min and max value or the 25th and 75th percentiles. Differences between patients with or without GCA were evaluated with the Mann-Whitney *U*-test. Categorical variables were tested with Fisher's exact test. The sensitivity and specificity (95% CI) of CDU were calculated using the clinical GCA diagnosis after 6 months as reference. Statistical analyses were carried out using the SPSS 27.0 for Windows software (IBM Corp., Armonk, NY, USA). Differences with *p*-values < 0.05 were considered statistically significant.

### Ethical considerations

The study was performed according to the Declaration of Helsinki, and the study protocol was approved by the Regional Ethical Board in Linköping (Decision number, 2013/33-31). Written informed consent for participation was not required for this study in accordance with the national legislation and the institutional requirements.

## Results

### Demographic and clinical features of patients with suspected GCA

In total, 107 patients without a previous diagnosis of GCA were included and examined with an extended CDU protocol. One patient who died within 6 months of the CDU examination was excluded from the final analysis. The median age (range) of the participants was 74 (48–92) years, and 68 (64%) were females and 33 (31%) received a clinical diagnosis of GCA based on an evaluation that was conducted ≥ 6 months after the CDU. The baseline characteristics of the patients are detailed in Table 1. Temporal artery abnormalities, jaw claudication, and symptoms of fatigue were seen more frequently in patients who received a GCA diagnosis, and these patients had higher levels of CRP and ESR and were older. The 1990 ACR criteria were fulfilled in 25 (76%) and the 2022 ACR/EULAR criteria in 31 (94%) of the patients with GCA (18, 25). Cranial symptoms (headache, jaw claudication, and/or vision disturbances) were noted for 25 patients (76%). The median (25th−75th percentile) duration of prednisolone treatment before CDU was 0 (0–1) days.

**Table 1:** Patients' characteristics and comparison between patients with and without giant cell arteritis.

**Patients' characteristics**	**Patients with GCA (*n* = 33)**	**Patients without GCA (*n* = 74)**	***p*-value**
Age, median (range) years	76 (63–92)	73 (44–89)	0.006
Female, *n* (%)	21 (64)	47 (64)	1.0
Smoking, *n* (%)	2 (6)	6 (8)	1.0
**Clinical characteristics**, ***n*** **(%)**			
New headache	23 (70)	38 (62)	0.093
Jaw claudication	12 (36)	3 (4)	< 0.0001
Reduced or lost vision	5 (18)	11 (15)	1.0
Double vision	1 (3)	1 (1)	0.52
Temporal artery abormalities^a^	20 (61)	21 (29)	0.0024
Joint pain	8 (24)	33 (45)	0.054
Fatigue	16 (49)	20 (28)	0.045
Loss of appetite	7 (21)	12 (16)	0.59
Weight loss > 2 kg	11 (33)	11 (15)	0.15
Temp > 38.5°C	2 (6)	1 (1)	0.22
**Laboratory findings** ^b^			
ESR, mm/h, median (range)	72 (15–119)	56 (2–106)	0.017
CRP, mg/L, median (range)	48 (4–225)	19 (4–173)	0.030
**Comorbidities**, ***n*** **(%)**			
Hypertension	21 (64)	40 (55)	0.40
Diabetes mellitus	13 (39)	14 (19)	0.031
Hyperlipidaemia	13 (39)	24 (32)	0.52
Myocardial infarction	4 (12)	7 (10)	0.74
Cerebrovascular disease	4 (12)	5 (7)	0.45
Peripheral artery disease	2 (6)	0 (0)	0.093

^a^Tenderness, pain, swelling or decreased pulsations.

^b^Erythrocyte sedimentation rate (ESR) and C-reactive protein (CRP) were measured before initiation of high-dose glucocorticoid treatment.

### High-frequency CDU and SMI for patients with suspected GCA

Of the 33 patients with GCA, CDU detected affection of the temporal artery in 31 (94%), facial artery in 17 (52%), axillary artery in 8 (24%), subclavian artery in 6 (18%), brachiocephalic artery in 1 (3%), and common carotid artery in 7 (21%). Vessel wall inflammation were restricted to the cranial vessels in 21 patients (64%), 2 (6%) had inflammation that was restricted to extra-cranial vessels, and 10 (30%) displayed a mixed phenotype, with inflammatory changes in both the cranial and extra-cranial arteries.

**Figure 1:**
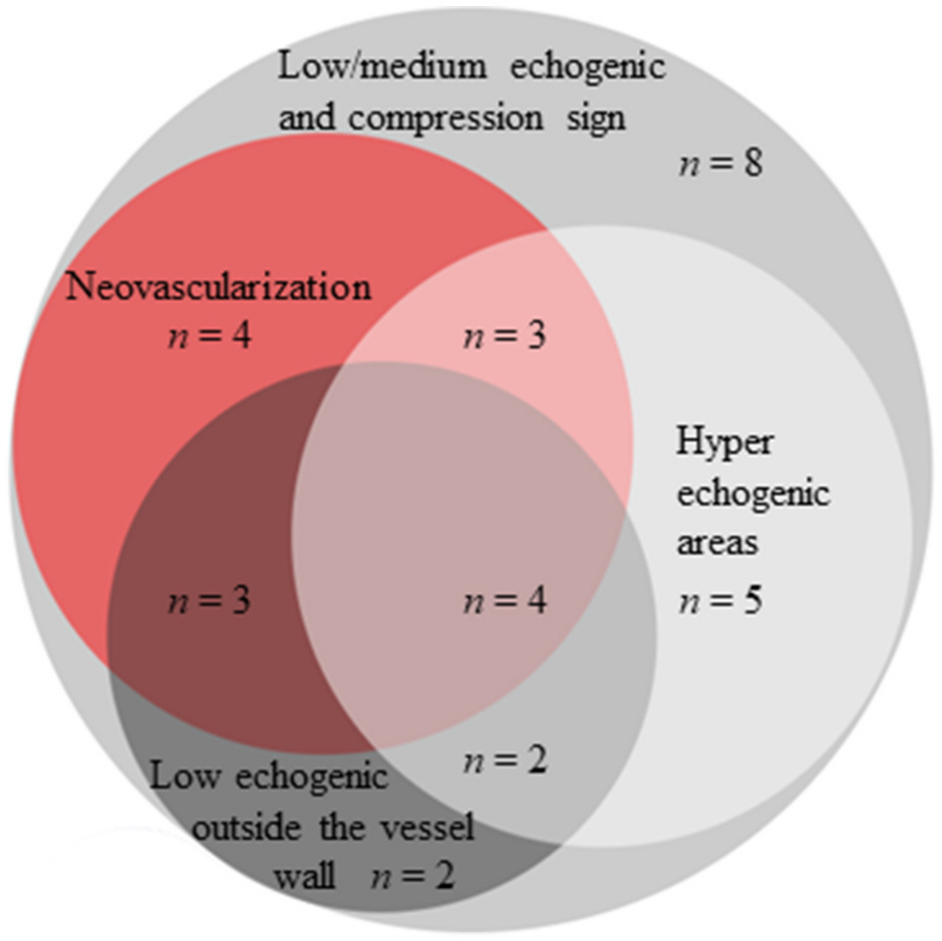
Morphological findings in the temporal arteries of patients with GCA using high-frequency ultrasound and SMI.

**Figure 2:**
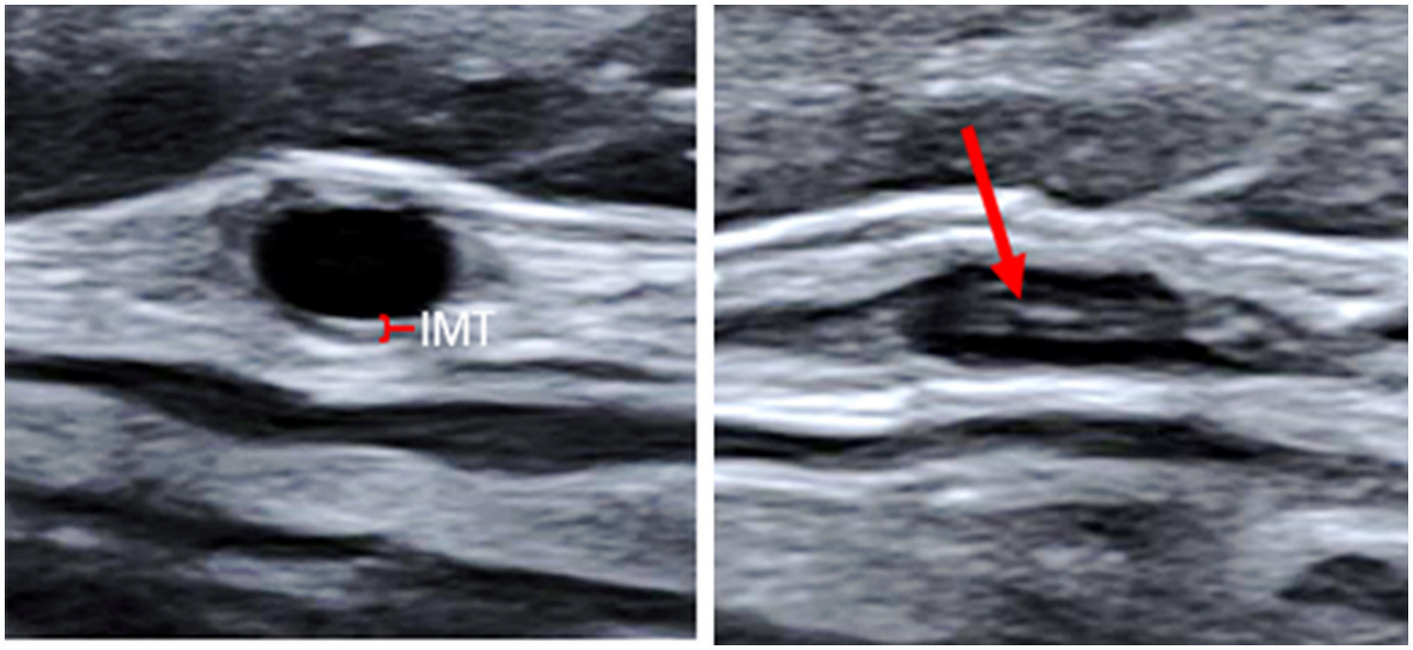
Transverse ultrasound images of the common superficial temporal artery. Non-compressed to the left (the red mark indicates the normal thin IMT), and compressed to the right. The high-echogenic part (red arrow) in the middle of the compressed vessel is the intima from the near and far wall pressed together. IMT, Intima-media thickness.

**Figure 3:**
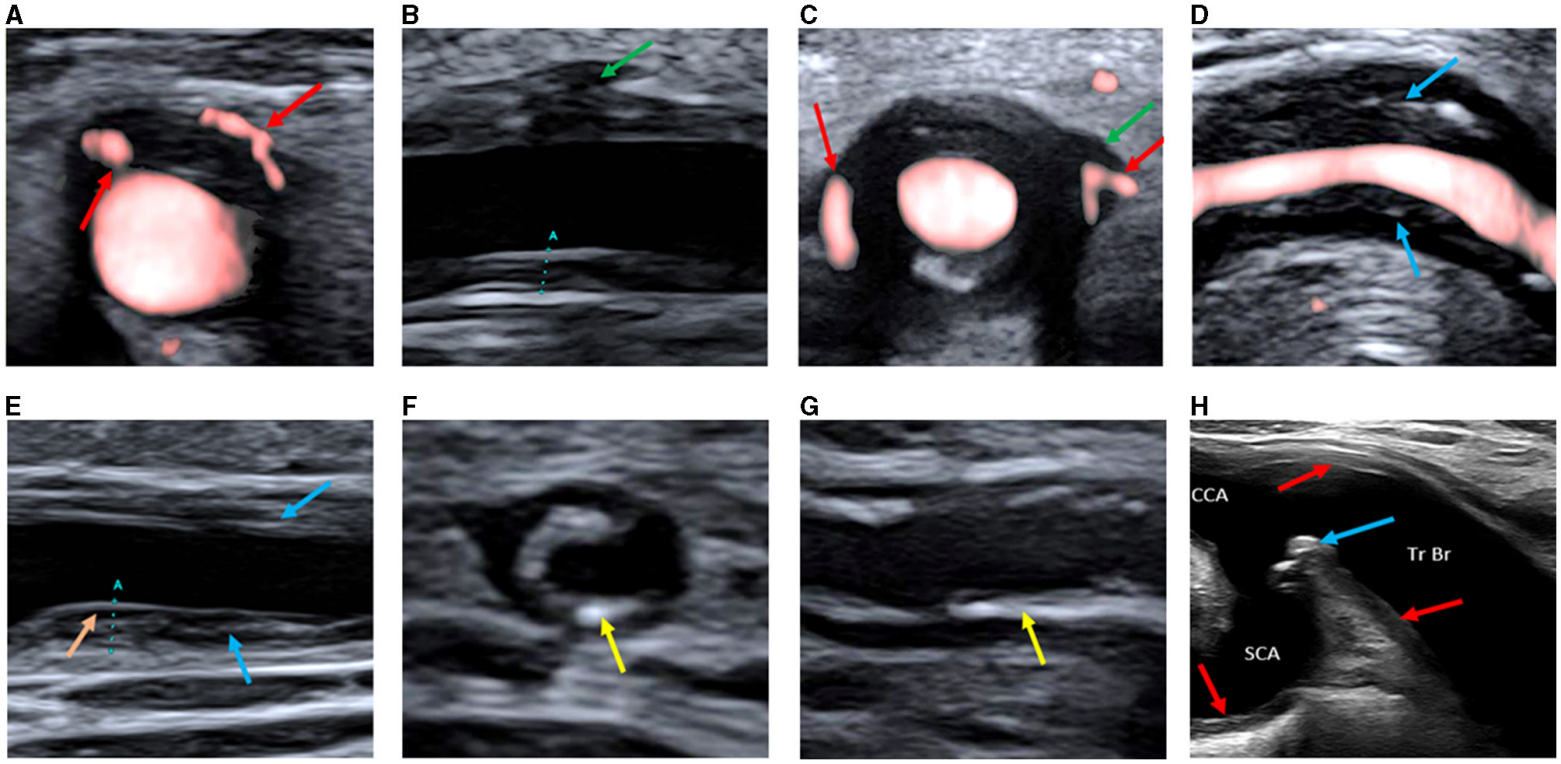
**(A)** Transverse ultrasound image of the common superficial temporal artery showing increased vessel wall thickness with hypoechogenicity and neovascularization in the wall (red arrows). **(B)** Longitudinal ultrasound image of the common superficial temporal artery showing a hypo-low-echogenic area outside the vessel wall (interpreted as inflammatory oedema, green arrow). The marker **(A)** is the IMT measurement. **(C)** Transverse ultrasound image of the common superficial temporal artery showing a hypo-low-echogenic area outside the vessel wall (interpreted as inflammatory oedema, green arrow) and neovascularization both in and outside the wall (red arrows). **(D)** Longitudinal ultrasound image of the facial artery showing increased vessel wall thickness with medium-echogenic areas (blue arrows), without neovascularization or hypo-echogenic areas outside the vessel wall. **(E)** Longitudinal ultrasound image of the common superficial temporal artery showing increased vessel wall thickness with medium-echogenic areas (blue arrows), without neovascularization or hypo-echogenic areas outside the vessel wall. A hypo-echogenic area interpreted as oedema below the intima layer is seen in the far wall (orange arrow). The marker **(A)** is the IMT measurement. **(F)** Transverse ultrasound image of the common superficial temporal artery showing increased vessel wall thickness with high-echogenicity areas (yellow arrows). **(G)** Longitudinal ultrasound image of the common superficial temporal artery showing increased vessel wall thickness with high-echogenicity areas (yellow arrow). **(H)** Ultrasound image of the brachiocephalic trunk and proximal parts of common carotid and subclavian artery, showing both inflammatory changes in the vessel walls (red arrows) and atherosclerotic plaque (blue arrow). SCA, subclavian artery; CCA, common carotid artery; Tr Br, brachiocephalic trunk; IMT; Intima-media thickness.

Based on the CDU protocol of the temporal arteries different morphological patterns could be distinguished as displayed in Figure 1. It is of note that the high resolution allows for the different layers of the vessel wall to be visualized. A consequence of this is that during compression of the temporal artery, the bright visible intima layers may be misinterpreted as a positive compression sign (Figure 2). Eighteen patients demonstrated increased IMT with low-medium echogenicity and a positive compression test together with neovascularization (Figure 3A) and/or low-medium-echogenic areas outside the vessel wall (interpreted as inflammatory oedema) (Figures 3B, C). Thirteen patients demonstrated increased IMT with low-medium echogenicity and a positive compression test without additional signs of neovascularization or low-medium echogenic areas outside the vessel wall (Figures 3D, E). Fourteen patients showed areas of high echogenicity in combination with a low- to medium-echogenic homogeneous wall thickening (Figures 3F, G). One patient also showed sub-intimal hypo-echogenic areas interpreted as oedema (Figure 3E). The IMT measurements and atherosclerotic burdens are shown in Table 2. Atherosclerotic plaques in the carotid arteries were detected in >80% of patients, with no differences between the groups. Figure 3H shows one patient who had both large vessel vasculitis and atherosclerotic plaque in the brachiocephalic artery.

**Table 2:** IMT and atherosclerotic plaques in patients with and without giant cell arteritis (GCA).

**Variable**	**Patients with GCA (*n* = 33)**	**Patients without GCA (*n* = 74)**	***p*-value**
**IMT, right**			
TA superficial, median (IQR)	0.55 (0.40–0.71)	0.20 (0.16–0.24)	< 0.0001
AxA, mm median (IQR)	0.70 (0.60–1.00)	0.65 (0.50–0.70)	0.007
SCA, mm median (IQR)	0.80 (0.70–1.03)	0.60 (0.50–0.80)	0.003
CCA, mm median (IQR)	0.80 (0.70–1.00)	0.80 (0.70–0.90)	0.091
**IMT, left**			
TA superficial, median (IQR)	0.59 (0.43–0.69)	0.20 (0.16–0.25)	< 0.0001
AxA, mm median (IQR)	0.70 (0.60–0.80)	0.60 (0.50–0.70)	0.12
SCA, mm median (IQR)	0.80 (0.65–0.95)	0.60 (0.50–0.70)	< 0.0001
CCA, mm median (IQR)	0.90 (0.80–1.10)	0.80 (0.70–1.00)	0.13
**Atherosclerotic plaque**			
Carotid area, *n* (%)	29 (88%)	61 (82%)	0.58

IQR, interquartile range; GCA, giant cell arteritis; IMT, intima-media thickness; AxA, axillary artery; SCA, subclavian artery; CCA, common carotid artery; TA, temporal artery.

Using SMI, neovascularization was detected in 14 patients (43%). Neovascularization was associated with more-extensive inflammation in terms of the numbers of affected cranial vessels, i.e., the common superficial temporal artery, its parietal and frontal branches and the facial artery, [6 (5.75–8) vs. 3 (2–6), *p* < 0.001], as well as a higher halo count [6 (4.75–7) vs. 3 (2–4), *p* = 0.005]. No significant differences were found regarding extra-cranial inflammation between patients with neovascularization and those without neovascularization [0 (0–2.25) vs. 0 (0–2), *p* = 0.53]. The CRP and ESR levels were also similar in patients with and without neovascularization [for CRP: 68 (27–89) vs. 38 (6–103), *p* = 0.27; and for ESR 73 (48–95) vs. 65 (59–90), *p* = 0.69]. Of all the patients diagnosed with GCA, 11 (33%) showed a low-medium echogenic area outside the vessel wall interpreted as oedema. No differences in the number of affected cranial vessels (*p* = 0.59), halo score (*p* = 0.85) or inflammatory markers such as CRP (*p* = 0.77) or ESR (*p* = 0.62) were detected between the GCA patients with and without oedema outside the vessel wall.

### CDU and diagnostic accuracy for patients with suspected GCA

CDU evaluation of the temporal arteries yielded diagnostic sensitivity and specificity values [95% confidence intervals (CI)] of 94% (80–99%) and 100% (89–100%), respectively. The sensitivity and specificity were unchanged after adding the facial artery. When the extra-cranial vessels, i.e., axillary, subclavian and common carotid arteries, were added the sensitivity and specificity values were 100% (89–100%) and 100% (95–100%), respectively.

### CDU for patients with a previous GCA diagnosis

Thirteen patients had a previous diagnosis of GCA and were admitted to CDU based on suspicion of inflammatory relapse (Table 3). Many of these patients had old lesions that were visualized with CDU. However, some of them were assessed long ago with old ultrasound equipment making it difficult to differentiate between new and old findings. Nonetheless, Case 8 in Table 3 is interesting because neovascularization together with low echogenic vessel wall swelling strongly suggests inflammatory activity (in a patient showing progression as well as regression of inflammatory wall changes in different vessels).

**Table 3:** Color duplex ultrasound (CDU) at follow-up of patients with previously established diagnosis of giant cell arteritis (GCA).

**No**	**Year of GCA diagnosis/ follow-up**	**CDU at follow-up**		**CRP/ESR at follow-up (mg/L)**	**Symptoms at follow-up**	**Steroids before CDU follow-up**	**Clinical relapse at evaluation**
		**Morphology/IMT**	**Active** ^a^				
1	2016/2020	TA: no compression sign, high echogenicity. FA: increased IMT, medium-high echogenicity.	No	8/8	Headache	2.5 mg	No^b^
2	2020/2021	TA: compression sign, low-medium echogenicity.	Active	140/117	CSx, hip pain	12.5 mg	Yes
3	2010/2022	TA and FA: compression sign, medium-high echogenicity. AxA: fibrotic stripes.	No?	4/87	CSx (weight loss)		No^c^
4	2019/2020	TA: medium-high echogenicity, borderline increased IMT.	No	4/10	Headache, chronic pain	20 mg	No^b^
5	2015/2021	AxA and SCA: fibrotic stripes.	No	4/34	CSx, dementia	10 mg	No
6	2015/2020	Right TA: high echogenicity, borderline increased IMT. Left TA:^d^	No?	10/80	Headache	50 mg	No^b^
7	2020/2021	Normal	No	9/34	None	30–40 mg	No
8	2021/2021	TA: decreased and increased IMT, low echogenicity and neovascularization. SCA and AxA: decreased IMT. FA: increased IMT.	Active	64/73	Stiff muscles	5 mg	Yes
9	2020/2022	Normal	No	25/60	None	0	No
10	2020/2021	Normal	No	4/6	Headache	5 mg	No^b^
11	2018/2021	SCA and CCA: medium echogenicity.	No?	4/12	Temporal discomfort	0	No
12	2020/2021	Normal	No	11/26	None	0	No
13	2017/2022	AxA, SCA, CCA: medium echogenicity, increasing IMT.	Active?	25/65	None	5 mg + TCZ	Yes

^a^Active inflammation of the vessel wall.

^b^Non-specific headache.

^c^Myeloma explained high ESR.

^d^Difficult to assess due to previous TAB.

CDU, color duplex ultrasound; TA, temporal artery; FA, facial artery; AxA, axillary artery; SCA, subclavian artery; CCA, common carotid artery; TCZ, Tocilizumab; IMT, Intima-Media Thickness, Csx; constitutional symptoms. Steroids implied Prednisolone in all cases.

## Discussion

The present study shows that modern ultrasound equipment, including high frequency transducers and software such as SMI, allows the detection of inflammatory neovascularization and facilitates the interpretation of morphological changes in temporal arteries. This technique, combined with our extended CDU protocol, that includes extra cranial-vessels, results in high diagnostic sensitivity for patients with suspected GCA.

Current recommendations regarding the diagnosis of GCA with ultrasound are based on the halo sign and the compression sign (2). Halo is traditionally referred to as a homogeneous, hypo- or iso-echoic wall thickening, and the compression sign is defined as a thickened arterial wall that remains visible upon transducer-imposed compression (26). However, the latest ultrasound equipment enables the detection of additional details of the vessel wall. The homogeneous halo observed with older equipment (Supplementary Figure 1) is replaced by an image that visualizes the different layers of the arterial wall. At compression of the artery, the intima layer can be distinguished from the media layer and simulate incomplete compression (a false-positive compression sign) to the unexperienced observer. The new technique also facilitates more-detailed localization of inflammatory oedemas, including sub-intimal and extra-vasal oedemas. Whether or not isolated histological oedemas outside the vessel wall can facilitate the diagnosis of active arteritis has been a matter of debate (27–29). However, we observed extra-vasal oedema together with other ultrasonographic signs of arteritis in 33% of the patients with GCA in the present cohort, but it is unclear if extra-vasal oedema in addition to other ultrasound findings indicates a higher degree of inflammation. In addition, the diagnostic relevance of sub-intimal oedema observed in one patient is unclear. This finding has, to the best of our knowledge, not been reported previously.

CDU has become an important tool in diagnosing GCA, and EULAR recommends ultrasound of the temporal and axillary arteries as the first imaging modality (2). Interestingly, a recent meta-analysis based on CDU examination that was restricted to the temporal arteries showed that the sensitivity for diagnosing GCA was higher in studies conducted after year 2010, as compared to studies conducted before year 2010 (71 vs. 63%) (4). More-recent studies, not included in the meta-analysis, have shown even higher diagnostic sensitivities in the range of 80–86% (5, 7, 30). While the reasons for this are probably multifactorial, it is important to consider the extensive development of the ultrasound technique that has occurred in recent years. In the present study using a 22-MHz probe for cranial examinations, the sensitivity obtained for CDU that was restricted to the temporal arteries was 94% [95% CI, (80–99)]. High-frequency probes are now used more commonly, whereas previous studies often used probes with lower frequencies (4). The importance of the CDU equipment has been examined by Noumegni et al. who compared images from 18- to 22-MHz probes and reported that in some cases the pathology could only be visualized by using the 22-MHz probe (31). Besides the development of the ultrasound technique, the increased number of examined arteries may also have contributed to the increased sensitivity of GCA diagnosis. Compared to when only temporal arteries were studied, our extended protocol increased the sensitivity from 94 to 100% (95% CI, 89–100).

New ultrasound imaging modalities, such as contrast-enhanced ultrasound (CEUS) and SMI, have been developed. Both modalities are able to visualize low-velocity blood flow in the vessel wall representing inflammatory neovascularization (8, 32). CEUS has been used to detect neovascularization in larger arteries, although it has proven difficult to use in smaller arteries such as the temporal vessels (32–34). Furthermore, CEUS requires intravenous injection and is time-consuming which means that its use in the clinical routine is problematic. In contrast, SMI is less time-consuming and can be used in multiple vascular areas without the use of injected contrast agents. In the present study, SMI was used in the temporal arteries, and neovascularization was detected in 43% of the patients with ultrasonographic signs of inflammation but not in those without inflammation. Patients with neovascularization displayed a more-extended inflammation when the affected cranial vessels, i.e., temporal and facial vessels, were enumerated. Furthermore, neovascularization was also associated with higher halo counts. Neovascularization in temporal arterial biopsies has been associated with a more-prominent systemic inflammatory response based on clinical data, including fever, weight loss, anemia and ESR as well as a higher level of infiltration of mononuclear inflammatory cells in the vessel wall (9, 10). Halo counts have also been shown to correlate positively with the levels of CRP (20). However, no differences in CRP or ESR were found in our patients with GCA regardless of the presence or absence of neovascularization. Nevertheless, this is the first study to apply SMI to consecutive GCA patients, and further research on SMI in GCA is warranted in relation to its role as a diagnostic, prognostic or monitoring tool for disease activity in both cranial and extra-cranial vessels.

Follow-up of disease activity in patients with GCA using CDU is challenging. Inflammation-induced vessel wall thickening may disappear or persist despite the arteritis being in clinical remission. If earlier CDU examinations are available, there may appear contradictory results showing both an increase and decrease of IMT in different vessels, and images derived using old and modern machines are often difficult to compare. Nevertheless, as was seen in 1 of the 13 patients with previously known GCA, hypo-echogenic vessel wall swelling combined with neovascularization in the temporal artery is a as sign of relapsing disease. CDU of this patient showed both progression and regression of IMT in different vessel areas, and the visualization of neovascularization facilitated the diagnosis of relapse. Recently an Ultrasonography Score (OGUS) was developed for monitoring disease activity in patients with GCA (35, 36). OGUS is quantitative score based on IMT measurements in the temporal artery and its branches, as well as in the axillary artery. Although quantitative scores, such as OGUS, most certainly will help clinicians to evaluate disease activity over time, concurrent increases and decreases in IMT across different vascular beds can complicate the overall interpretation. In such cases, SMI, with its relatively straightforward morphological interpretation, may serve as a valuable complementary tool.

Some limitations are worth noting. Although the present study used consecutive recruitment, the design was retrospective in that the patients were cared for by different rheumatologists using different clinical protocols. Nevertheless, a strength of the study was the strictly standardized CDU protocol performed by one experienced vascular technologist. PET was only conducted in cases where it was clinically indicated, and comparison of the PET and CDU results was not planned prospectively and could not be performed retrospectively. The present study was also limited by its sample size and its relative predominance toward a cranial phenotype of the disease, which may affect the calculated distribution among the various GCA phenotypes.

## Conclusions

Modern ultrasound technique has facilitated visualization of morphological changes in the arterial wall of patients with GCA. We show for the first time that SMI can be used to visualize neovascularization in the temporal arteries as a sign of inflammation in the vessel wall and that neovascularization seems to be related to a more-widespread cranial disease. Nevertheless, further prospective studies are required to evaluate whether SMI can provide prognostic information and to determine its role in the monitoring of disease activity.

## Data availability statement

The raw data supporting the conclusions of this article will be made available by the authors, without undue reservation.

## Ethics statement

The studies involving humans were approved by the Regional Ethical Board in Linköping (Dnr. 2013/33-31). The studies were conducted in accordance with the local legislation and institutional requirements. The Ethics Committee/Institutional Review Board waived the requirement of written informed consent for participation from the participants or the participants' legal guardians/next of kin because written informed consent for participation was not required for this study in accordance with the national legislation and the institutional requirements.

## Author contributions

JS: Writing – original draft. CSv: Writing – review & editing. PE: Writing – review & editing. CSj: Writing – review & editing. HZ: Writing – review & editing.
