# Supplementary material for: High-frequency ultrasound with superb microvascular imaging: a potential tool for ultrasound assessment in patients with giant cell arteritis
Source: Front Med (Lausanne). 2024 Jul 10;11:1431385. doi: 10.3389/fmed.2024.1431385 (PMC11266178; doi:10.3389/fmed.2024.1431385)
Supplement: Supplementary file 1 [file Image_1.pdf]

Supplementary Material

High-frequency ultrasound with superb microvascular imaging: a potential tool for ultrasound assessment in patients with giant cell arteritis

Johan Skoog*, Christina Svensson, Per Eriksson, Christopher Sjöwall, Helene Zachrisson

Correspondence: johan.skoog@liu.se

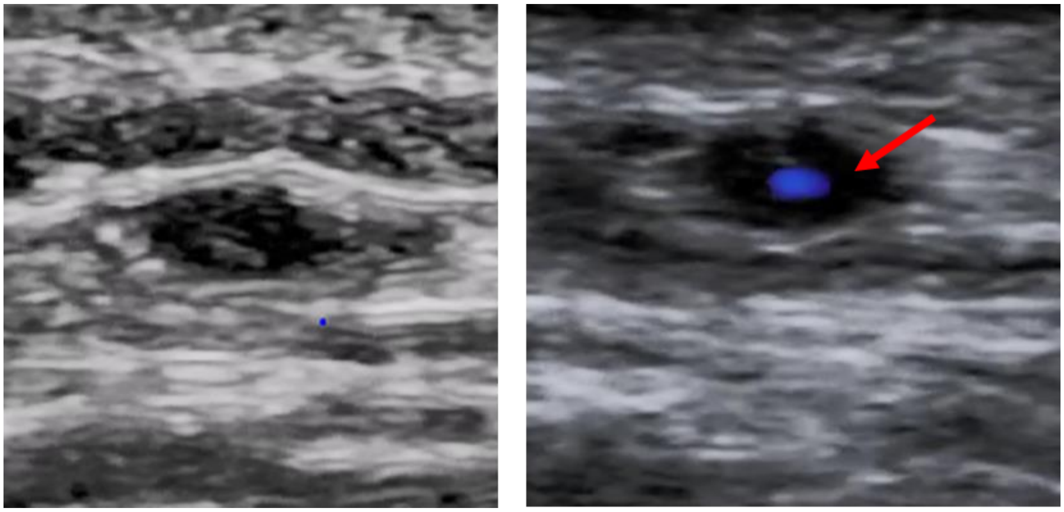

Supplementary Figure 1. Ultrasound images of the common superficial temporal artery with the previous (>5 years old) generation of ultrasound equipment. Measurement of the intima-media thickness and evaluation of the vessel wall were not possible. The finding was described as a halo and non-compressible artery. Red arrow: hypo-echogenic halo.
